# Natural Language Processing for Analysing Patient and Caregiver Experiences of Dementia with Lewy Bodies

**DOI:** 10.1002/alz70861_108463

**Published:** 2025-12-23

**Authors:** Judith Rose Harrison, Rania Shamshul Baharain, Paul C Donaghy, Stuart Maitland

**Affiliations:** ^1^ Translational and Clinical Research Institute, Faculty of Medical Sciences, Newcastle University, Newcastle upon Tyne, England UK; ^2^ Newcastle University, Malaysia, Newcastle UK; ^3^ Newcastle University, Newcastle upon Tyne UK; ^4^ Translational and Clinical Research Institute Faculty of Medical Sciences Newcastle University, Newcastle UK

## Abstract

**Background:**

Dementia with Lewy Bodies (DLB) is a neurodegenerative disorder with complex and distressing symptoms, often leading to misdiagnosis and delayed care. While clinical assessments are essential, they may not capture the full breadth of symptomatology experienced by patients and caregivers. Social media, particularly platforms like Reddit, offers patients and caregivers a space to share their challenges in an organic, unstructured way, which can reveal critical insights into symptomatology and caregiving burdens. This study explores how Natural Language Processing (NLP) can be used to analyse this real‐world data on DLB symptoms and caregiving challenges.

**Method:**

We applied NLP techniques to a large dataset of unstructured text from Reddit discussions. Using the Reddit for Researchers framework, we accessed anonymised comments from the r/Dementia subreddit (44,000 users). Predefined keyword groups focusing on themes related to DLB symptoms, caregiving challenges, medical management, and prognosis were used to query relevant posts. We employed a range of NLP methods, including sentiment analysis to gauge the emotional tone of the posts, and topic modeling (via non‐negative matrix factorization) to identify and categorise key themes. We analysed the frequency of different symptom mentions, caregivers' reported challenges, and perceptions of DLB treatment and management.

**Result:**

A total of 36,125 comments were analysed, resulting in the identification of several prominent sub‐themes. These included descriptions of core DLB symptoms, such as cognitive fluctuations, hallucinations, and motor symptoms, as well as the emotional and physical burden on caregivers. Additionally, analysis revealed frequent mentions of challenges in obtaining a proper diagnosis and navigating healthcare systems, suggesting areas where clinical awareness may need improvement. Topic modeling found caregivers discussing handling hallucinations, managing side effects of medications and planning end of life care. There were also frequent mentions of the emotional impact of DLB, with many caregivers expressing feelings of isolation and frustration with the lack of timely, accurate diagnosis.

**Conclusion:**

Applying NLP to social media discussions uncovers valuable insights into the challenges faced by DLB patients and caregivers. This study demonstrates how integrating social media insights into clinical research can provide a deeper understanding of DLB, potentially enhancing diagnosis and treatment strategies.

